# Hierarchical Dirichlet process model for gene expression clustering

**DOI:** 10.1186/1687-4153-2013-5

**Published:** 2013-04-12

**Authors:** Liming Wang, Xiaodong Wang

**Affiliations:** 1 Department of Electrical & Computer Engineering, Duke University, Durham, NC, 27708, USA; 2 Department of Electrical Engineering, Columbia University, New York, NY, 10027, USA

## Abstract

Clustering is an important data processing tool for interpreting microarray data and genomic network inference. In this article, we propose a clustering algorithm based on the hierarchical Dirichlet processes (HDP). The HDP clustering introduces a hierarchical structure in the statistical model which captures the hierarchical features prevalent in biological data such as the gene express data. We develop a Gibbs sampling algorithm based on the Chinese restaurant metaphor for the HDP clustering. We apply the proposed HDP algorithm to both regulatory network segmentation and gene expression clustering. The HDP algorithm is shown to outperform several popular clustering algorithms by revealing the underlying hierarchical structure of the data. For the yeast cell cycle data, we compare the HDP result to the standard result and show that the HDP algorithm provides more information and reduces the unnecessary clustering fragments.

## 1 Introduction

The microarray technology has enabled the possibility to monitor the expression levels of thousands of genes in parallel under various conditions [1]. Due to the high-volume nature of the microarray data, one often needs certain algorithms to investigate the gene functions, regulation relations, etc. Clustering is considered to be an important tool for analyzing the biological data [2-4]. The aim of clustering is to group the data into disjoint subsets, where in each subset the data show certain similarities to each other. In particular, for microarray data, genes in each clustered group exhibit correlated expression patterns under various experiments.

Several clustering methods have been proposed, most of which are distance-based algorithms. That is, a distance is first defined for clustering purpose and then the clusters are formed based on the distances of the data. Typical algorithms in this category include the K-means algorithm [5] and the self-organizing map (SOM) algorithm [6]. These algorithms are based on simple rules, and they often suffer from robustness issue, i.e., they are sensitive to noise which is extensive in biological data [7]. For example, the SOM algorithm requires user to provide number of clusters in advance. Hence, incorrect estimation of the parameter may provide wrong result.

Another important category of clustering methods is the model-based algorithms. These algorithms employ a statistical approach to model the structure of clusters. Specifically, data are assumed to be generated by some mixture distribution. Each component of the mixture corresponds to a cluster. Usually, the parameters of the mixture distribution are estimated by the EM algorithm [8]. The finite-mixture model [9-11] assumes that the number of mixture components is finite and the number can be estimated using the Bayesian information criterion [12] or the Akaike information criterion [13]. However, since the estimation of the number of clusters and the estimation of the mixture parameters are performed separately, the finite-mixture model may be sensitive to the different choices of the number of clusters [14].

The infinite-mixture model has been proposed to cope with the above sensitivity problem of the finite-mixture model. This model does not assume a specific number of components and is primarily based on the Dirichlet processes [15,16]. The clustering process can equivalently be viewed as a Chinese restaurant process [17], where the data are considered as customers entering a restaurant. Each component corresponds to a table with infinite capacity. A new customer joins a table according to the current assignment of seats.

Hierarchical clustering (HC) is yet another more advanced approach especially for biological data [18], which groups together the data with similar features based on the underlying hierarchical structure. The biological data often exhibit hierarchical structure, e.g., one cluster may highly be overlapped or could be embedded into another cluster [19]. If such hierarchical structure is ignored, the clustering result may contain many fragmental clusters which could have been combined together. Hence, for biological data, such HC has its advantages to many traditional clustering algorithms. The performances of such HC algorithms depend highly on the quality of the data and the specific agglomerative or divisive ways the algorithms use for combining clusters.

**Figure 1:**
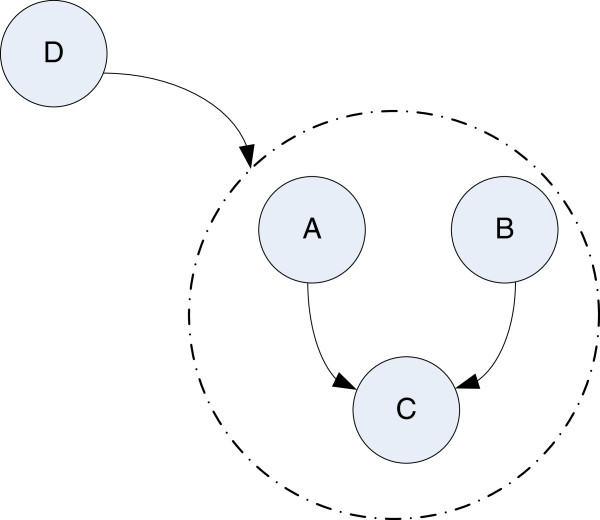
Illustration of gene hierarchical structures in microarray data.

Traditional clustering algorithms for microarray data usually assign each gene with a feature vector formed by the expressions in different experiments. The clustering is carried out for these vectors. It is well known that many genes share different levels of functionalities [20]. The resemblances of different genes are commonly represented at different levels of perspectives, e.g., at the cluster level instead of individual gene level. In other words, The relationships among different genes may vary during different experiments. In Figure 1, we illustrate the gene hierarchical structures for microarray data. Genes group A and B may show close relationship to genes group C in some experiments. While the genes group D shows correlations to groups A, B, and C in other experiments. The group D obviously has a hierarchical relationships to other gene groups. In this case, we desire to have a HC algorithm recognizing the gene resemblances not at the single gene level but at the higher cluster level, to avoid unnecessary fragmental clusters that impede the proper interpretation of the biological information. Such a HC algorithm may also provide new information by taking the hierarchical similarities into account.

In this article, we propose a model-based clustering algorithm for gene expression data based on the hierarchical Dirichlet process (HDP) [21]. The HDP model incorporates the merits of both the infinite-mixture model and the HC. The hierarchical structure is introduced to allow sharing data among related clusters. On the other hand, the model uses the Dirichlet processes as the non-parametric Bayesian prior, which do not assume a fixed number of clusters *a priori*.

The remainder of the article is organized as follows. In Section 2, we introduce some necessary mathematical background and formulate the HC problem as a statistical inference problem. In Section 3, we derive a Gibbs sampler-based inference algorithm based on the Chinese restaurant metaphor of the HDP model. In Section 4, we provide experimental results of the proposed HDP algorithm for two applications, regulatory network segmentation and gene expression clustering. Finally, Section 5 concludes the article.

## 2 System model and problem formulation

As in any model-based clustering method, it is assumed that the gene expression data are random samples from some underlying distributions. All data in one cluster are generated by the same distribution. For most existing clustering algorithms, each gene is associated with a vector containing the expressions in all experiments. The clustering of the genes is based on their vectors. However, such approach ignores the fact that genes may show different functionalities under various experiment conditions, i.e., different clusters may be formed under different experiments. In order to cope with this phenomenon, we treat each expression separately. More specifically, we allow different expressions of the same individual gene to be generated by different statistical models.

Suppose that for the mircoarray data, there are *N* genes in total. For each gene, we conduct *M* experiments. Let *g*_*ji*_ denote the expression of the *i*th gene in the *j*th experiment, 1≤*i*≤*N*, and 1≤*j*≤*M*. For each *g*_*ji*_, we associate a latent membership variable *z*_*ji*_, which indicates the cluster membership of *g*_*ji*_. That is, if genes *i* and *i*^′^ are in the same cluster under the conditions of experiments *j* and *j*^′^, we have $z_{\text{ji}}=z_{j^{\prime }i^{\prime }}$. Note that *z*_*ji*_ is supported on a countable set such as N or Z. For each *g*_*ji*_, we associate a coefficient $\theta_{z_{\text{ji}}}$, whose index is determined by its membership variable *z*_*ji*_. In order to have a Bayesian approach, we also assume that each coefficient *θ*_*k*_ is drawn independently from a prior distribution *G*_0_

(1) $$\begin{array}{llll} \theta_{k}∼G_{0}, & & & \end{array}$$

where *k* is determined by *z*_*ji*_.

The membership variable **z**={*z*_*ji*_}_*j*,*i*_ has a discrete joint distribution

(2) $$\begin{array}{llll} z∼\mathrm{\Pi .} & & & \end{array}$$

Note that in this article, the bold-face letter always refers to a set formed by the elements with specified indices.

We assume that each *g*_*ji*_ is drawn independently from a distribution $F(\theta_{z_{\text{ji}}})$

(3) $$\begin{array}{llll} g_{\text{ji}}∼F\left(\theta_{z_{\text{ji}}}\right), & & & \end{array}$$

where $\theta_{z_{\text{ji}}}$ is a coefficient associated with *g*_*ji*_ and *F* is a distribution family such as the Gaussian distribution family. In summary, we have the following model for the expression data

(4) $$\begin{array}{cccc} \theta_{k} & ∼G_{0} & & \\ z & ∼\Pi & & \\ g_{\text{ji}}|z_{\text{ji}},\theta_{k} & ∼F\left(\theta_{z_{\text{ji}}}\right). & & \end{array}$$

The above model is a relatively general one which can induce many previous models. For example, in all Bayesian approaches, all variables are assigned with proper priors. It is very popular to use the mixture model as the prior, which models the data generated by a mixture of distributions, e.g., a linear combination of a family of distributions such as Gaussian distributions. Each cluster is generated by one component in the mixture distribution given the membership variable [14]. The above approach corresponds to our model if we assume that *Π* is finitely supported and *F* is Gaussian.

The aim for clustering is to determine the posterior probability of the latent membership variables given the observed gene expressions

(5) $$\begin{array}{llll} P(z|g), & & & \end{array}$$

where **g**={*g*_*ji*_}_*j*,*i*_.

As a clustering algorithm, the final result is given in the forms of clusters. Each gene has to be assigned to one and only one cluster. Once we have the inference result in (5), we can apply the maximum *a posterior* criterion to obtain an estimate of membership variable ${\hat{z}}_{\cdot i}$ for the *i*th gene as

(6) $$\begin{array}{l} {\hat{z}}_{\cdot i}=\arg_{a}\;\max\underset{j}{\sum }P(z_{\text{ji}}=a|g). \end{array}$$

We note that in case one is interested in finding other related clusters for one gene, we can simply use the inferred distribution to membership variable to obtain this information.

### 2.1 Dirichlet processes and infinite mixture model

Instead of assuming a fixed number of clusters *a priori*, one can assume infinite number of clusters to avoid the estimation accuracy problem on the number of clusters as we mentioned earlier. Correspondingly in (4), the prior *Π* is an infinite discrete distribution. Again as in the Bayesian fashion, we will introduce priors for all parameters. The Dirichlet process is one such prior. It can be viewed as a random measure [15], i.e., the domain of this process (viewed as a measure) is a collection of probability measures. In this section, we will give a brief introduction to the Dirichlet process which serves as the vital prior part in our HDP model.

Recall that the Dirichlet distribution $D(u_{1},\ldots ,u_{K})$ of order *K* on a (*K*−1)-simplex in $R^{K-1}$ with parameter *u*_1_,…,*u*_*K*_ is given by the following probability density function

(7) $$D(x_{1},\ldots ,x_{K-1};u_{1},\ldots ,u_{K})=\frac{\Gamma \left(\sum_{i=1}^{K}u_{i}\right)}{\prod_{i=1}^{K}\Gamma (u_{i})}\overset{K}{\underset{i=1}{\prod }}{x_{i}}^{u_{i}-1}$$

where $\sum_{i=1}^{K}x_{i}=1,u_{i}>0,i=1,\ldots ,K,$ and *Γ*(·) is the Gamma function. Since every point in the domain is a discrete probability measure, the Dirichlet distribution is a random measure in the finite discrete probability space.

The Dirichlet processes are the generalization of the Dirichlet distribution into the continuous space. There are various constructive or non-constructive definitions of Dirichlet processes. For simplicity, we use the following non-constructive definition.

Let (*X*,*σ*,*μ*_0_) be a probability space. A Dirichlet process *D*(*α*_0_,*μ*_0_) with parameter *α*_0_>0 is defined as a random measure: for any non-trivial finite partition (*χ*_1_,…,*χ*_*r*_) of *X* with *χ*_*i*_∈*σ*, we have the random variable

(8) $$(G(\chi_{1}),\ldots ,G(\chi_{r}))∼D(\alpha_{0}\mu_{0}(\chi_{1}),\ldots ,\alpha_{0}\mu_{0}(\chi_{r})),$$

where G is drawn from *D*(*α*_0_,*μ*_0_).

The Dirichlet processes can be characterized in various ways [15] such as the stick-breaking construction [22] and the Chinese restaurant process [23]. The Chinese restaurant process serves as a visualized characterization of the Dirichlet process.

Let *x*_1_,*x*_2_,… be a sequence of random variables drawn from the Dirichlet process *D*(*α*_0_,*μ*_0_). Although we do not have the explicit formula for *D*, we would like to know the conditional probability of *x*_*i*_ given *x*_1_,…,*x*_*i*−1_. In the Chinese restaurant model, the data can be viewed as customers sequentially entering a restaurant with infinite number of tables. Each table corresponds to a cluster with unlimited capacity. Each customer *x*_*i*_ entering the restaurant will join in the table already taken with equal probability. In addition, the new customer may sit in a new table with probability proportional to *α*_0_. Tables that have already been occupied by customers tend to gain more and more customers.

One remarkable property of the Dirichlet process is that although it is generated by a continuous process, it is discrete (countably many) almost surely [15]. In other words, almost every sample distribution drawn from the Dirichlet process is a discrete distribution. As a consequence, the Dirichlet process is suitable to serve as a non-parametric prior of the infinite mixture model.

The Dirichlet mixture model uses the Dirichlet process as a prior. The model in (4) can then be represented as follows:

(9) $$\begin{array}{cccc} g_{\text{ji}}|z_{\text{ji}},\theta_{k}∼F(\theta_{z_{\text{ji}}}); & & & \end{array}$$

*θ*_*k*_ is generated by the measure *μ*_0_

(10) $$\begin{array}{c} \theta_{k}∼\mu_{0}; \end{array}$$

{*z*_*ji*_} is generated by a Dirichlet process *D*(*α*_0_,*μ*_0_)

(11) $$\begin{array}{c} \{z_{\text{ji}}\}∼D(\alpha_{0},\mu_{0}). \end{array}$$

Recall that *D*(*α*_0_,*μ*_0_) is discrete almost everywhere, which corresponds to the indices of the clusters.

### 2.2 HDP model

Biological data such as the expression data often exhibit hierarchical structures. For example, although clusters can be formed based on similarities, some clusters may still share certain similarities among themselves at different levels of perspectives. Within one cluster, the genes may share similar features. But on the level of clusters, one cluster may share some similar feature with some other clusters. Many traditional clustering algorithms typically fail to recognize such hierarchical information and are not able to group these similar clusters into a new cluster, producing many fragments in the final clustering result. As a consequence, it is difficult to interpret the functionalities and meanings of these fragments. Therefore, it is desirable to have an algorithm that is able to cluster among clusters. In other words, the algorithm should be able to cluster based on multiple features at different levels. In order to capture the hierarchical structure feature of the gene expressions, we now introduce the hierarchical model to allow clustering at different levels. The clustering algorithm based on the hierarchical model not only reduces the number of cluster fragments, but also may reveal more details about the unknown functionalities of certain genes as the clusters sharing multiple features.

Recall that in the statistical model (11), the clustering effect is induced by the Dirichlet process *D*(*α*_0_,*μ*_0_). If we need to take into account different level of clusters, it is natural to introduce a prior with clustering effect to the base measure *μ*_0_. Again in this case, the Dirichlet process can serve as such prior. The intuition is that given the base measure, the clustering effect is represented through a Dirichlet process on the single gene level. By the Dirichlet process assumption on the base measure, the base measure also exhibits the clustering effect, which leads to clustering at cluster level. We simply set the prior to the base measure *μ*_0_ as

(12) $$\begin{array}{cccc} \mu_{0}∼D_{1}(\alpha_{1},\mu_{1}), & & & \end{array}$$

where *D*_1_(*α*_1_,*μ*_1_) is another Dirichlet process. In this article, we use the same letter for the measure, the distribution it induces, and the corresponding density function as long as it is clear from the context. Moreover, we could extend the hierarchies to as many levels as we wish at the expense of complexity of the inference algorithm. The desired number of hierarchies can be determined by the prior biological knowledge. In this article, we focus on a two-level hierarchy.

As a remark, we would like to point out the connection and difference on the “hierarchy” in the proposed HDP method and traditional HC [4]. Both the HDP and HC algorithms can provide HC results. The hierarchy in the HDP method is manifested by the Chinese restaurant process which will be introduced later, where the data sit in the same table can be viewed as the first level and all tables sharing the same dish can be viewed as the second level. While the hierarchy in the HC is obtained by merging existing clusters based on their distances. However, its specific merging strategy is heuristic and is irreversible for those merged clusters. Hierarchy formed in this fashion often may not reflect the true structure in the data since various hierarchical structures can be formed by choosing different distance metrics. However, the HDP algorithm captures the hierarchical structure at the model level. The merging is carried out automatically during the inference. Therefore, it naturally takes the hierarchy into consideration.

In summary, we have the following HDP model for the data:

(13) $$\begin{array}{rcll} \mu_{0} & ∼ & D_{1}(\alpha_{1},\mu_{1}) & \\ \{z_{\text{ji}}\}|\mu_{o},\alpha_{0} & ∼ & D(\alpha_{0},\mu_{0}) & \\ \alpha_{0},\alpha_{1} & ∼ & \Gamma (a,b) & \\ \theta_{k} & ∼ & \mu_{1} & \\ g_{\text{ji}}|z_{\text{ji}},\theta_{k} & ∼ & F(\theta_{z_{\text{ji}}}), & \end{array}$$

where *a* and *b* are some fixed constants. We assume that *F* and *μ*_1_ are conjugate priors. In this article, *F* is assumed to be the Gaussian distribution and *μ*_1_ is the inverse Gamma distribution.

## 3 Inference algorithm

It is intractable to get the closed-form solution to the inference problem (5). In this section, we develop a Gibbs sampling algorithm for estimating the posterior distribution in (5). At each iteration *l*, we draw a sample $z_{\text{ji}}^{\left(l\right)}$ sequentially from the distribution:

(14) $$P\left(z_{\text{ji}}^{\left(l\right)}|z_{11}^{\left(l\right)},z_{12}^{\left(l\right)},\ldots ,z_{j(i-1)}^{\left(l\right)},z_{j(i+1)}^{\left(l-1\right)},\ldots ,z_{\text{MN}}^{\left(l-1\right)},g\right).$$

Under regularity conditions, the distribution of ${\left\{z_{\text{ji}}^{\left(l\right)}\right\}}_{j,i}$ will converge to the true posterior distribution in (5) [24]. The proposed Gibbs sampling algorithm is similar to the HDP inference algorithm proposed in [21], since both the Gibbs algorithms use the Chinese restaurant metaphor which we will elaborate later. However, because of the differences in modeling, we still need to provide details for the inference algorithm based on our model.

### 3.1 Chinese restaurant metaphor

The Chinese restaurant model [23] is a visualized characterization for interpreting the Dirichlet process. Because there is no explicit formula to describe the Dirichlet process, we will employ the Chinese restaurant model for HDP inference instead of directly computing the posterior distribution in (5). We refer to [23,25] for the proof and other details of the equivalence between the Chinese restaurant metaphor and the Dirichlet processes.

**Figure 2:**
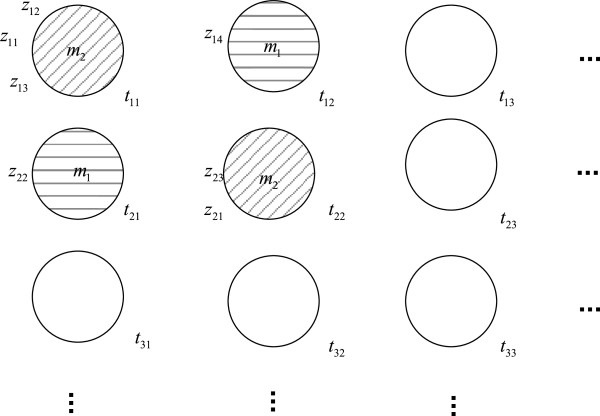
**Illustration of the Chinese restaurant metaphor.** Tables of the same pattern are grouped into the same cluster.

In the Chinese restaurant metaphor for the HDP model (13), we view {*z*_*ji*_} as customers entering a restaurant sequentially. The restaurant has infinite number of rows and columns of tables which are labeled by *t*_*ji*_. Each *z*_*ji*_ will associate to one and only one table in the *j*th row. We use *ϕ*(*z*_*ji*_) to denote the column index of the table in the *j*th row taken by *z*_*ji*_, i.e., *z*_*ji*_ will sit at table $t_{\mathrm{j\phi}(z_{\text{ji}})}$. If it is clear from the context, we will use *ϕ*_*ji*_ in short for *ϕ*(*z*_*ji*_). The index of the random variable *θ*_*k*_ in (13) is characterized by a menu containing various dishes. Each table picks one and only one dish from the menus {*m*_*k*_}_*k*=1,2,…_, which are drawn independently from the base measure *μ*_1_. *g*_*ji*_ is drawn independently according to the dish it chooses through the distribution *F*(·) as in (13). We denote *λ*(*t*_*ji*_) as the index of the dish taken by table *t*_*ji*_, i.e., table *t*_*ji*_ chooses dish $m_{\lambda (t_{\text{ji}})}$. As before, we may write *λ*_*ji*_ in short of *λ*(*t*_*ji*_). In summary, customer *z*_*ji*_ will sit at table $t_{j\phi_{\text{ji}}}$ and enjoy dish $m_{\lambda_{j\phi_{\text{ji}}}}$. The HDP is reflected in this metaphor such that the customers choose the tables as well as the dishes in a Dirichlet process fashion. The customers sitting at the same table are classified into one cluster. Moreover, the customers sitting at different tables but ordering the same dish will also be clustered into the same group. Hence, the clustering effect is performed at the cluster level, i.e., we allow “clustering among clusters”. In Figure 2, we show an illustration of the Chinese restaurant metaphor. The different patterns of shades represent different clusters. We also introduce two useful counter variables: *c*_*ji*_ denotes the number of customers sitting at table *t*_*ji*_; *d*_*jk*_ counts the number of tables in row *j* serving dish *m*_*k*_.

Using the Chinese restaurant metaphor, instead of inferring *z*_*ji*_, we can directly infer *ϕ*_*ji*_ and *λ*_*ji*_. The membership variable *z*_*ji*_ is completely determined by $\lambda (t_{\mathrm{j\phi}(z_{\text{ji}})})$. That is, $z_{\text{ji}}=z_{j^{\prime }i^{\prime }}$ if and only if $\lambda (t_{\mathrm{j\phi}(z_{\text{ji}})})=\lambda (t_{\mathrm{j\phi}(z_{j^{\prime }i^{\prime }})})$. As we pointed out before, the specific values of the membership variable *z*_*ji*_ are not relevant to the clustering as long as *z*_*ji*_ is supported on a countable set. Hence, we could simply let

(15) $$\begin{array}{c} z_{\text{ji}}=\lambda \left(t_{\mathrm{j\phi}(z_{\text{ji}})}\right). \end{array}$$

According to [25], we have the following conditional probabilities for the HDP model

(16) $$\begin{array}{ll} \phi_{\text{ji}}|\phi_{j1},\ldots ,\phi_{\text{ji}-1},\alpha_{0},\mu_{0}∼ & \;\overset{\sum_{k}d_{\text{jk}}}{\underset{m=1}{\sum }}\frac{c_{\text{jm}}}{i-1+\alpha_{0}}\delta_{t_{j\phi_{\text{ji}}}}\\ \;+\frac{\alpha_{0}}{i-1+\alpha_{0}}\mu_{0}, \end{array}$$

where $\underset{k}{\sum }d_{\text{jk}}$ calculates the number of tables taken in the *r*th row and *δ*_(·)_ is the Kronecker delta function. The interpretation of (16) is that customer *z*_*ji*_ chooses a table already taken with equal probability. In addition, *z*_*ji*_ may choose a new table with probability proportional to *α*_0_.

By the hierarchical assumption, the distribution of the dish chosen at an occupied table is another Dirichlet process. We have the following conditional distribution of the dishes

(17) $$\begin{array}{ll} \lambda_{j\phi_{\text{ji}}}|\lambda_{1\phi_{11}},\ldots ,\lambda_{j\phi_{j(i-1)}},\alpha_{1},\mu_{1}∼ & \;\overset{K_{\text{ji}}}{\underset{k=1}{\sum }}\frac{\sum_{j}d_{\text{jk}}}{\sum_{\text{jk}}d_{\text{jk}}+\alpha_{1}}\delta_{m_{k}}\\ \;+\frac{\alpha_{1}}{\sum_{\text{jk}}d_{\text{jk}}+\alpha_{1}}\mu_{1}, \end{array}$$

where $\sum_{j}d_{\text{jk}}$ counts the number of tables serving dish *m*_*k*_; $\sum_{\text{jk}}d_{\text{jk}}$ counts the number of tables serving dishes; *K*_*ji*_ denotes the net number of dishes served till *λ*_*ji*_’s coming by counting only once each dish that has been served multiple times.

### 3.2 A Gibbs sampler for HDP inference

Instead of sampling the posterior probability in (5), we will sample ***ϕ***={*ϕ*_11_,*ϕ*_12_,…} and ***λ***={*λ*_11_,*λ*_12_,…} from the following posterior distribution

(18) $$\begin{array}{l} P(\phi ,\lambda |g). \end{array}$$

We can calculate the related conditional probabilities as follows.

If *a* is a value that has been taken before, the conditional probability of *ϕ*_*ji*_=*a* is given by

(19) $$P\left(\phi_{\text{ji}}=a|\phi_{\text{ji}}^{c},\lambda ,\theta ,\alpha_{1},\alpha_{0},\mu_{1},g\right)\propto c_{\text{ja}}f_{\lambda_{\text{ja}}}(g_{\text{ji}}|g_{\text{ji}}^{c}),$$

where ***θ***={*θ*_*ji*_}_*j*,*i*_ and ***λ***={*λ*_*ji*_}_*j*,*i*_. The superscript *c* denotes the complement of the variables in its category, i.e., $g_{\text{ji}}^{c}={\left\{g_{j^{\prime }i^{\prime }}\right\}}_{\left(j^{\prime },i^{\prime })\neq (j,i\right)}$ and $\phi_{\text{ji}}^{c}={\left\{\phi_{j^{\prime }i^{\prime }}\right\}}_{\left(j^{\prime },i^{\prime })\neq (j,i\right)}$. $f_{\lambda_{\text{ja}}}\left(g_{\text{ji}}|g_{\text{ji}}^{c}\right)$ denotes the conditional density of *g*_*ji*_ given all other data generated according to menu $m_{\lambda_{\text{ja}}}$, which can be calculated as

(20) $$f_{\lambda_{\text{ja}}}\left(g_{\text{ji}}|g_{\text{ji}}^{c}\right)=\frac{\int \underset{\lambda_{j^{\prime }\phi_{j^{\prime }i^{\prime }}}=\lambda_{\text{ja}}}{\prod }F(g_{j^{\prime }i^{\prime }}|\theta )\mu_{1}(\theta )\mathrm{d휃}}{\int \underset{j^{\prime }i^{\prime }\neq \text{ji},\lambda_{j^{\prime }\phi_{j^{\prime }i^{\prime }}}=\lambda_{\text{ja}}}{\prod }F(g_{j^{\prime }i^{\prime }}|\theta )\mu_{1}(\theta )\mathrm{d휃}}.$$

The numerator of (20) is the joint density of the data which are generated by the same dish. By the assumption that $g_{j^{\prime }i^{\prime }}$ are conditionally independent given the chosen dish, we have the conditional density of the data in the product form. The denominator is the joint density excluding the specific *g*_*ji*_ term. The integrals in (20) can either be calculated using the numerical method or using the Monte Carlo integration. For example, in order to calculate the following integral $\int_{a}^{b}f(x)p(x)\text{dx}$, where *p*(*x*) is a density function, we can draw samples *x*_1_,*x*_2_,…,*x*_*n*_ from *p*(*x*) and approximate the integral by $\int_{a}^{b}f(x)p(x)\text{dx}=E_{p(x)}[f(x)]\approx \frac{1}{n}\sum_{i=1}^{n}f(x_{i})$. To calculate (20), we view *μ*_1_(·) as *p*(·) and $F(g_{j^{\prime }i^{\prime }}|\cdot )$ as *f*(·).

On the other hand, if *a* is a new value then we have

(21) $$\begin{array}{lll} & P\left(\phi_{\text{ji}}=a|\phi_{\text{ji}}^{c},\lambda ,\theta ,\alpha_{1},\alpha_{0},g\right)\propto \alpha_{0}\; & \;\\ & \left[\overset{K_{\text{ja}}}{\underset{k=1}{\sum }}\frac{\sum_{j}d_{\text{jk}}}{\sum_{\text{jk}}d_{\text{jk}}+\alpha_{1}}f_{k}\left(g_{\text{ji}}|g_{\text{ji}}^{c}\right)\right)\; & \;\\ & \;\left(+\frac{\alpha_{1}}{\sum_{\text{jk}}d_{\text{jk}}+\alpha_{1}}\int F(g_{\text{ji}}|\theta )\mu_{1}(\theta )\mathrm{d\theta}\right].\; \end{array}$$

We also have the following conditional probabilities for *λ*_*ji*_. If *a* is used before, we have

(22) $$\;P\left(\lambda_{j\phi_{\text{ji}}}=a|\phi ,\lambda_{j\phi_{\text{ji}}}^{c},\theta ,\alpha_{1},\alpha_{0},g\right)\propto \left(\underset{j}{\sum }d_{\text{ja}}\right)f_{a}\left(g_{\text{ji}}|g_{\text{ji}}^{c}\right);$$

otherwise we have

(23) $$\;P\left(\lambda_{j\phi_{\text{ji}}}=a|\phi ,\lambda_{j\phi_{\text{ji}}}^{c},\theta ,\alpha_{1},\alpha_{0},g\right)\propto \alpha_{1}\int F(g_{\text{ji}}|\theta )\mu_{1}(\theta )\mathrm{d\theta .}$$

The derivations of (19), (21), (22), and (23) are given in Appendix.

Before we present the Gibbs sampling algorithm, we recall the Metropolis–Hastings (M–H) algorithm [26] for drawing samples from a target distribution whose density function *f*(*x*) is only known up to a scaling factor, i.e., *f*(*x*)∝*p*(*x*). To draw samples from *f*(*x*), we make use of some fixed conditional distribution *q*(*x*_2_|*x*_1_) that satisfies *q*(*x*_2_|*x*_1_)=*q*(*x*_1_|*x*_2_), ∀*x*_1_,*x*_2_. The M–H algorithm proceeds as follows. 

•Start with an arbitrary value *x*_0_ with *p*(*x*_0_)>0.

•For *l*=1,2,… 

Given the previous sample *x*_*l*−1_, draw a candidate sample *x*^⋆^ from *q*(*x*^⋆^|*x*_*l*−1_).

Calculate $\beta =\frac{p(x^{⋆})}{p(x_{l-1})}$. If *β*≥1 then accept the candidate and let *x*_*l*_=*x*^⋆^. Otherwise accept it with probability *β*, or reject it and accept the previous sample with probability 1−*β*.

After a “burn-in” period, say *l*_0_, the samples ${\left\{x_{l}\right\}}_{l>l_{0}}$ follow the distribution *f*(*x*).

We now summarize the Gibbs sampling algorithm for the HDP inference as follows. 

•Initialization: randomly assign the indices $\phi^{\left(0\right)}=\left\{\phi_{11}^{\left(0\right)},\phi_{12}^{\left(0\right)},\ldots \right\}$ and $\lambda^{\left(0\right)}=\left\{\lambda_{11}^{\left(0\right)},\lambda_{12}^{\left(0\right)},\ldots \right\}$. Note that once we have all the indices, the counters {*c*_*ji*_} and {*d*_*jk*_} are also determined.

•For *l*=1,2,…,*l*_0_+*L*, 

Draw samples of $\left\{\phi_{\text{ji}}^{\left(l\right)}\right\}$ from their posteriors

(24) $$P\left(\phi_{\text{ji}}^{\left(l\right)}=a|\phi_{\text{ji}}^{(l-1)c},\lambda^{\left(l-1\right)},\alpha_{1}^{\left(l-1\right)},\alpha_{0}^{\left(l-1\right)},g\right)$$

given by (19) and (21) using the M–H algorithm. We view the probability in (24) as the target density and choose *q*(·|·) to be a distribution supported on N. For example, we can use $q(i|j)=\frac{j}{{\left(j+1\right)}^{i}}$, i,j∈N.

Draw samples of $\left\{\lambda_{j\phi_{\text{ji}}^{\left(l\right)}}^{\left(l\right)}\right\}$ from their posteriors

(25) $$P\left(\lambda_{j\phi_{\text{ji}}^{\left(l\right)}}^{\left(l\right)}=a|\phi^{\left(l\right)},\lambda_{j\phi_{\text{ji}}^{\left(l\right)}}^{(l-1)c},\alpha_{1}^{\left(l-1\right)},\alpha_{0}^{\left(l-1\right)},g\right)$$

given by (22) and (23) using M–H algorithm. We view the probability in (25) as the target density and use *q*(·|·) as specified in the previous step.

Since *P*(*α*_0_|***ϕ***,***λ***,*α*_1_,**g**)=*P*(*α*_0_) and *P*(*α*_1_|***ϕ***,***λ***,*α*_0_,**g**)=*P*(*α*_1_), simply draw samples of $\alpha_{0}^{\left(l\right)}$ and $\alpha_{1}^{\left(l\right)}$ from their prior Gamma distributions.

•Using the samples after the “burn-in” period ${\left\{\phi^{\left(l\right)},\lambda^{\left(l\right)}\right\}}_{l=l_{0}+1}^{l_{0}+L}$ to calculate $\hat{P}(\phi ,\lambda |g)$, which is given by

(26) $$\;\hat{P}\left(\phi_{\text{ji}}=a,\lambda_{j\phi_{\text{ji}}}=b\right)=\frac{\sum_{l=l_{0}+1}^{l_{0}+L}1\left\{\phi_{\text{ji}}^{\left(l\right)}=a,\lambda_{j\phi_{\text{ji}}^{\left(l\right)}}^{\left(l\right)}=b\right\}}{L},$$

•where **1**(·) is the indicator function. Determine the membership distribution *P*(**z**|**g**) from the inferred joint distribution $\hat{P}(\phi ,\lambda |g)$ by $P(z_{\text{ji}}=a|g)=\underset{b}{\sum }\hat{P}(\lambda_{\text{jb}}=a|g,\phi_{\text{ji}}=b)\hat{P}(\phi_{\text{ji}}=b|g)$.

•Calculate the estimation of clustering index ${\hat{z}}_{\cdot i}$ for the *i*th gene by ${\hat{z}}_{\cdot i}=\underset{a}{\arg}\max\;\sum_{j}P(z_{\text{ji}}=a|g)$.

### 3.3 A numerical example

In this section, we provide a simple numerical example to illustrate the proposed Gibbs sampler. Let us consider the case *N*=*M*=2, i.e., there are 2 genes and 2 experiments. Assume that the expressions are as *g*_11_=0,*g*_12_=1,*g*_21_=−1, and *g*_22_=2. We assume $\mu_{1}(\theta )∼N(0,1)$ and $F(g_{\text{ji}}|\theta )∼N(\theta ,1)$. For initialization, we set $\phi_{11}^{\left(0\right)}=1,\phi_{12}^{\left(0\right)}=2,\phi_{21}^{\left(0\right)}=3,\phi_{22}^{\left(0\right)}=4$; $\lambda_{1\phi_{11}^{\left(0\right)}}^{\left(0\right)}=1,\lambda_{1\phi_{12}^{\left(0\right)}}^{\left(0\right)}=1,\lambda_{2\phi_{21}^{\left(0\right)}}^{\left(0\right)}=2,\lambda_{2\phi_{22}^{\left(0\right)}}^{\left(0\right)}=2,$ and *α*0(0)=*α*1(0)=1.

We first show how to draw sample from $P\left(\phi_{11}^{\left(1\right)}|\phi_{11}^{(0)c},\right)$$\left(\lambda^{\left(0\right)},\alpha_{1}^{\left(0\right)},\alpha_{0}^{\left(0\right)},g\right)$ by the M–H algorithm. Given the initial value, assume that *q*(·|·) returns *ϕ*_11_=3 as a candidate sample. By (19), we have $P\left(\phi_{11}^{\left(1\right)}=1|\phi_{11}^{(0)c},\lambda^{\left(0\right)},\alpha_{1}^{\left(0\right)},\right)$$\left(\alpha_{0}^{\left(0\right)},g\right)\propto c_{11}f_{\lambda_{11}}\left(g_{11}|g_{11}^{c}\right)$, where *c*_11_=1 and *λ*_11_=1. We also have

(27) $$\begin{array}{lll} f_{1}\left(g_{11}|g_{11}^{c}\right) & =\frac{\int \underset{\lambda_{j^{\prime }\phi_{j^{\prime }i^{\prime }}}=1}{\prod }F(g_{j^{\prime }i^{\prime }}|\theta )\mu_{1}(\theta )\mathrm{d\theta}}{\int \underset{(j^{\prime },i^{\prime })\neq (1,1),\lambda_{j^{\prime }\phi_{j^{\prime }i^{\prime }}}=1}{\prod }F(g_{j^{\prime }i^{\prime }}|\theta )\mu_{1}(\theta )\mathrm{d\theta}}\; & \;\\ & =\frac{\int F(g_{11}|\theta )F(g_{12}|\theta )\mu_{1}(\theta )\mathrm{d\theta}}{\int F(g_{12}|\theta )\mu_{1}(\theta )\mathrm{d\theta}}\approx 0.22971.\; \end{array}$$

Note that the above integral can be calculated either numerically or by using the Monte Carlo integration method.

By (21) and using the specific values of the variables, we obtain

(28) $$\begin{array}{lll} & P\left(\overset{\left(1\right)}{\underset{11}{\phi }}=3|\phi_{11}^{(0)c},\lambda^{\left(0\right)},\alpha_{1}^{\left(0\right)},\alpha_{0}^{\left(0\right)},g\right)\; & \;\\ & \propto \alpha_{0}\left[\overset{K_{11}}{\underset{k=1}{\sum }}\frac{\sum_{j}d_{\text{jk}}}{\sum_{\text{jk}}d_{\text{jk}}+\alpha_{1}}f_{k}\left(g_{11}|g_{11}^{c}\right)\right)\; & \;\\ & \;\;\left(+\frac{\alpha_{1}}{\sum_{\text{jk}}d_{\text{jk}}+\alpha_{1}}\int F(g_{11}|\theta )\mu_{1}(\theta )\mathrm{d\theta}\right]\; \end{array}$$

with *K*_11_=1, $\sum_{j}d_{j1}=2$, $\sum_{\text{jk}}d_{\text{jk}}=4$, *α*_0_=*α*_1_=1. Plugging in these values, we have

(29) $$\begin{array}{lll} & \;P\left(\phi_{11}^{\left(1\right)}=3|\phi_{11}^{(0)c},\lambda^{\left(0\right)},\alpha_{1}^{\left(0\right)},\alpha_{0}^{\left(0\right)},g\right)\; & \;\\ & \;\propto \frac{2}{5}f_{1}\left(g_{11}|g_{11}^{c}\right)+\frac{1}{5}\int F(g_{11}|\theta )\mu_{1}(\theta )\mathrm{d\theta}\approx 0.1483.\; \end{array}$$

Since $\beta =\frac{0.1483}{0.22971}\approx 0.6456<1$, we should accept this candidate sample *ϕ*_11_=3 with a probability of 0.6456. After the burn-in period, say the sample returned by the M–H algorithm is *ϕ*_11_=4, then we update $\phi_{11}^{\left(1\right)}=4$ and move on to draw samples of the remaining variables *ϕ*_12_, *ϕ*_21_, and *ϕ*_22_.

Assuming that we obtain samples of ***ϕ***^(1)^ as $\phi_{11}^{\left(1\right)}=4,\phi_{12}^{\left(1\right)}=1,\phi_{21}^{\left(1\right)}=1,\phi_{22}^{\left(1\right)}=2$. We next draw the sample ***λ***^(1)^. Given the initial value $\lambda_{1\phi_{11}^{\left(1\right)}}=1$ and *q*(·|·) returns $\lambda_{1\phi_{11}^{\left(1\right)}}=3$ as a candidate sample. By (22), we obtain $P\left(\lambda_{1\phi_{11}^{\left(1\right)}}^{\left(1\right)}=1|\phi^{\left(1\right)},\lambda_{1\phi_{11}^{\left(1\right)}}^{(0)c},\alpha_{1}^{\left(0\right)},\alpha_{0}^{\left(0\right)},g\right)\propto \left(\underset{j}{\sum }d_{j1}\right)f_{1}\left(g_{11}|g_{11}^{c}\right)$. Furthermore, we have $\sum_{j}d_{j1}=2$ and $f_{1}\left(g_{11}|g_{11}^{c}\right)\approx 0.22971$ as calculated before.

By (23), we obtain $P\left(\lambda_{1\phi_{11}}^{\left(1\right)}=3|\phi^{\left(1\right)},\lambda_{1\phi_{11}}^{(0)c},\alpha_{1}^{\left(0\right)},\alpha_{0}^{\left(0\right)},g\right)\propto \alpha_{1}\int F(g_{11}|\theta )\mu_{1}(\theta )\mathrm{d\theta}$. Moreover, we have *α*_1_=1 and $\int F(g_{11}|\theta )\mu_{1}(\theta )\mathrm{d\theta}\approx 0.28208$ as calculated before. So we have $\beta =\frac{0.28208}{2*0.22971}\approx 0.614<1$. After the burn-in period, assume that the M–H algorithm returns a sample $\lambda_{1\phi_{11}^{\left(1\right)}}=2$, then update $\lambda_{1\phi_{11}^{\left(1\right)}}^{\left(1\right)}=2$ and move on to sample the remaining *λ* variables as well as *α*_0_ and *α*_1_.

After the burn-in period of the whole Gibbs sampler, we can calculate the posterior joint distribution *P*(***ϕ***,***λ***|**g**) from the samples and determine the clusters following the last two steps in the proposed Gibbs sampling algorithm.

## 4 Experimental results

The HDP clustering algorithm proposed in this article can be employed for gene expression analysis or as a segmentation algorithm for gene regulatory network inference. In this section, we first introduce two performance measures for clustering, the Rand Index (RI) [27] and the Silhouette Index (SI) [28]. We compare the HDP algorithm to the support vector machine (SVM) algorithm for network segmentation on synthetic data. We then conduct various experiments on both synthetic and real datasets including the AD400 datasets [29], the yeast galactose datasets [30], yeast sporulation datasets [31], human fibroblasts serum datasets [32], and yeast cell cycle data [33]. We compare the HDP algorithm to the Latent Dirichlet allocation (LDA), MCLUST, SVM, K-means, Bayesian Infinite Mixture Clustering (BIMC) the HC [4,14,34-37] based on the performance measures and the functional relationships.

### 4.1 Performance measures

In order to evaluate the clustering result, we utilize two measures: RI [27] and SI [28]. The first index is used when a ground truth is known in *priori* and the second index is to measure the performance without any knowledge of the ground truth.

The RI is a measure of agreement between two clustering results. It takes a value between 0 and 1. The higher is the score, the higher agreements it indicates.

Let *A* denote the datasets with a total number of *n* elements. Given two clustering results *X*={*X*_1_,…,*X*_*S*_} and *Y*={*Y*_1_,…,*Y*_*T*_} of *A*, i.e., $A=\overset{S}{\underset{i=1}{⋃}}X_{i}=\overset{T}{\underset{j=1}{⋃}}Y_{j}$ and $X_{i}⋂X_{j}=\emptyset$, $Y_{i}⋂Y_{j}=\emptyset$ for *i*≠*j*. For any pair of elements (*a*,*b*) in *A*, we say they are in the same set under a clustering result if *a* and *b* are in the same cluster. Otherwise we say they are in different sets. Note that there are totally n2 pairs of elements. We define the following four counting numbers: *Z*_1_ denotes the number of pairs that are both in the same set in *X* and *Y*; *Z*_2_ denotes the number of pairs that are both in different sets in *X* and *Y*; *Z*_3_ denotes the number of pairs that are in the same set in *X* and in different sets in *Y*; and *Z*_4_ denotes the number of pairs that are in different sets in *X* and in the same set in *Y*. The RI is then given by

(30) $$\begin{array}{cccc} \text{RI}=\frac{Z_{1}+Z_{2}}{Z_{1}+Z_{2}+Z_{3}+Z_{4}}. & & & \end{array}$$

Due to the lack of the ground truth in most real applications, we utilize the SI to evaluate the clustering performance. The SI is a measure by calculating the average width of all data points, which reflects the compactness of the clustering. Let *x* denote the average distance between a point *p* in a cluster and all other points within that cluster. Let *y* be the minimum average distance between *p* and other clusters. The Silhouette distance for *p* is defined as

(31) $$\begin{array}{llll} s(p)=\frac{y-x}{\max\{x,y\}}. & & & \end{array}$$

The SI is the average Silhouette distance among all data points. The value of SI lies in [−1,1] and higher score indicates better performance.

### 4.2 Network segmentation on synthetic data

In regulatory network inference, due to the large size of the network, it is often useful to perform a network segmentation. The segmented sub-networks usually have much less number of nodes than the original network, leading to faster and more accurate analysis of the original network [38]. Clustering algorithms can be employed for such segmentation purpose. However, traditional clustering algorithms often provide segmentation results either too fine or too coarse, i.e., the resulting sub-networks either contain too few genes or two many genes. In addition, the hierarchical structure of the network cannot be discovered by those algorithms. Thanks to its hierarchical model assumption, the HDP algorithm can provide better segmentation results. We demonstrate the segmentation application of HDP on a synthetic network and compare to the SVM algorithm which is widely used for clustering and segmentation.

**Figure 3:**
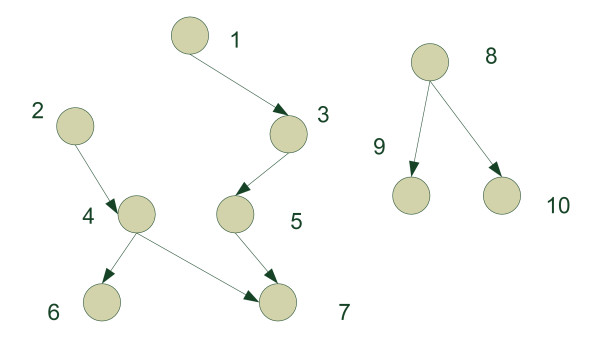
The synthetic network structure.

The network under consideration is shown in Figure 3. We assume that the distributions for all nodes are Gaussian. The directed links indicate that the parent nodes are the priors of the child nodes. Disconnected nodes are mutually independent. We generate the data in the following way. Nodes 1, 2, and 8 are generated independently by Gaussian distributions of unit variance with means 1, 2, and 3, respectively. Nodes 3, 4, 5, 6, 9, and 10 are generated independently by unit variance Gaussian distributions with means determined by their respective parent nodes. Node 7 is generated by a Gaussian distribution with mean determined by node 4 and variance determined by absolute value of node 5. The network contains two isolated segments with one segment containing nodes 1–7 and the other containing nodes 8–10. The HDP algorithm is applied to this network and segments the network into three clusters. Nodes 2, 4, 6 form one cluster; nodes 1, 3, 5, 7 form another cluster; and nodes 8, 9, 10 form the third one. The SVM algorithm on the other hand produces two clusters, one containing nodes 1–7 and the other containing nodes 8–10. As one can see, the network obviously contains two hierarchies in the left segment, i.e., nodes 1–7 of the network. The SVM fails to recognize the hierarchies and provides a result coarser than that given by the HDP algorithm.

### 4.3 AD400 data

The AD400 is a synthetic dataset proposed in [29], which is used to evaluate the clustering algorithm performance. The dataset is constituted by 400 genes with 10 time points. As the ground truth, the AD400 dataset has 10 clusters with each one containing 40 genes.

For randomized algorithms as LDA, BIMC, HDP, we average the results over 20 runs of the algorithms. We compare the HDP algorithm to other widely used algorithms such as LDA, SVM, MCLUST, K-means, BIMC, and HC. The results are presented in Table 1. As we can see, the HDP algorithm has the similar performance of the MCLUST algorithm. While the HDP generally performs better than other widely used algorithms.

**Table 1:** Clustering performance of LDA, SVM, MCLUST, K-means, HC, and HDP on the AD400 data

**Algorithm**	**RI**	**SI**	**Number of clusters**
LDA	0.931	0.553	10.0
SVM	0.929	0.493	11
MCLUST	0.942	0.583	10
K-means	0.895	0.457	10
HC	0.916	0.348	9
BIMC	0.935	0.571	10.0
HDP	0.947	0.577	10.0

### 4.4 Yeast galactose data

**Figure 4:**
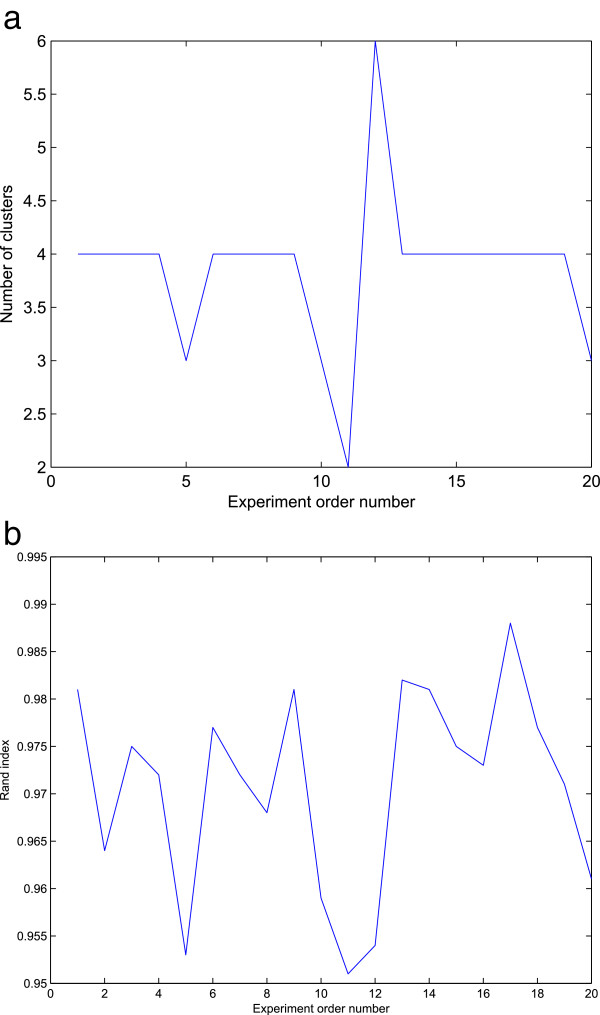
**Plots of the HDP results in 20 experiments. (a)** Plot of number of clusters in 20 experiments. **(b)** Plot of rand index in 20 experiments.

We conduct experiment on the yeast galactose data, which consists of 205 genes. The true number of clusters based on the functional categories is 4 [39]. We calculate the RI index between different clustering results to the result in [39], which is regarded as the standard benchmark. The LDA model is a generative probabilistic model for document classifications [34], which also uses Dirichlet distribution as a prior. We adapt the LDA model to the yeast galactose data to compare the proposed HDP algorithm. Since the LDA and HDP methods are randomized algorithms, we run the algorithms 20 times and use the average for the final score. In Figure 4, we illustrate the performances of each experiments for the HDP method. The performances of the algorithms under consideration are listed in Table 2.

**Table 2:** Clustering performance of LDA, MCLUST, SVM, and HDP on the yeast galactose data

**Algorithm**	**Rand index**	**Number of clusters**
LDA	0.942	6.3
SVM	0.954	5
MCLUST	0.903	9
HDP	0.973	3.8

It is seen that the HDP algorithm performs the best among the three algorithms. Unlike the MCLUST and LDA algorithms which produce more clusters than 4, the average number of clusters given by the HDP algorithm is very closed to the “true” value 4. Compared to the SVM method, the HDP algorithm produces a result that is more similar to the “ground truth”, i.e., with the highest RI value.

### 4.5 Yeast sporulation data

The yeast sporulation dataset consists of 6,118 genes with 7 times points which were obtained during the sporulation process [31]. We pre-processed the dataset by applying a logarithmic transform and removing the data whose expression levels did not have significant changes. After the pre-process, the data have 513 genes left. In Table 3, we compare the HDP clustering result to LDA, MCLUST, K-Means, BIMC, and HC. For randomized algorithms such as LDA, BIMC, and HDP, we average the scores by running the algorithm 20 times.

**Table 3:** Clustering performance of LDA, MCLUST, K-means, HC, BIMC, and HDP on the yeast sporulation data

**Algorithm**	**SI**	**Number of clusters**
LDA	0.586	6.2
MCLUST	0.577	6
K-Means	0.324	8
HC	0.392	7
BIMC	0.592	6.1
HDP	0.673	6.0

From Table 3, we can see that the HDP has the highest SI score. It suggests that the clustering results provided by HDP are more compact and less separated than results from other algorithms. The K-means and HC algorithm suggest higher number of clusters. However, their SI scores indicate that their clusters are not as tight as other algorithms.

### 4.6 Human fibroblasts serum data

The human fibroblasts serum data consists of 8,613 genes with 12 time points [32]. Again a logarithmic transform has been applied to the data and genes without significant changes have been removed. The remaining dataset has 532 genes.

In Table 4, we show the performance of the HDP algorithm and other various algorithms. It has been shown that the clustering results by the HDP algorithm are the compactest among those algorithms. The LDA algorithm suggests 9.4 clusters with the lowest SI score, which indicates that some of its clusters can be further tightened. HC provides a result consisting of five clusters. However, the SI score of the HC result is not the highest, which suggests its clustering may not be well formed.

**Table 4:** Clustering performance of LDA, MCLUST, K-means, HC, BIMC, and HDP on the human fibroblasts serum data

**Algorithm**	**SI**	**Number of clusters**
LDA	0.298	9.4
MCLUST	0.382	6
K-Means	0.324	7
HC	0.313	5
BIMC	0.418	7.3
HDP	0.452	6.4

### 4.7 Yeast cell cycle data

**Figure 5:**
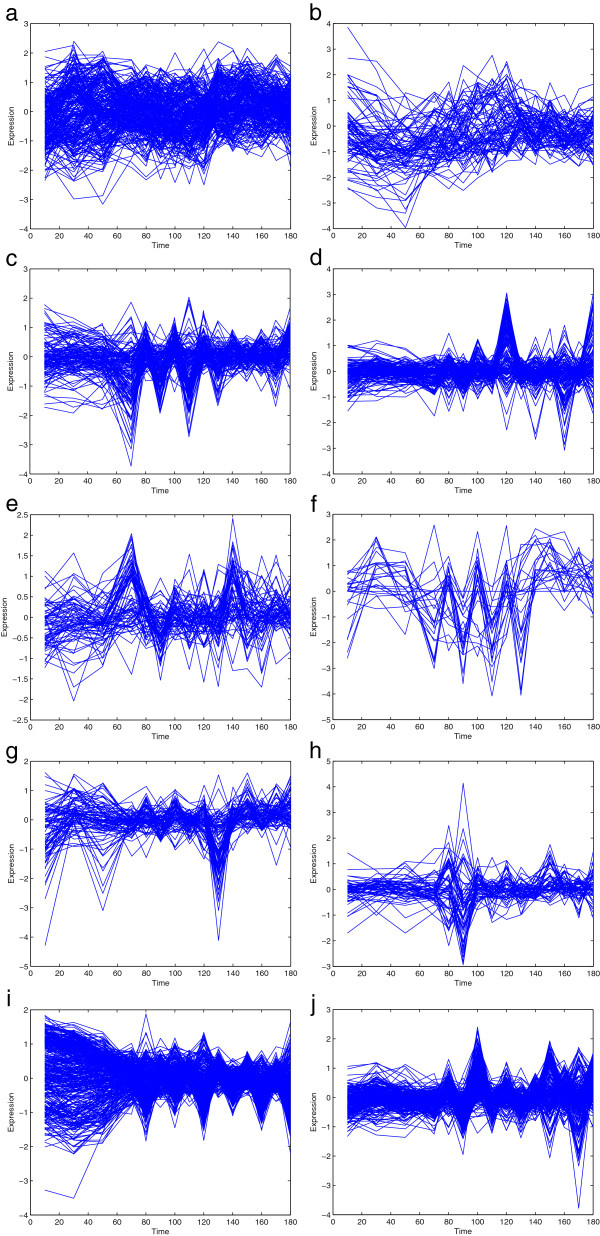
**Plots of all HDP clusters for yeast cell cycle data. (a)** Plot of Cluster 1, containing 261 genes. **(b)** Plot of Cluster 2, containing 86 genes. **(c)** Plot of Cluster 3, containing 135 genes. **(d)** Plot of Cluster 4, containing 144 genes. **(e)** Plot of Cluster 5, containing 76 genes. **(f)** Plot of Cluster 6, containing 25 genes. **(g)** Plot of Cluster 7, containing 88 genes. **(h)** Plot of Cluster 8, containing 60 genes. **(i)** Plot of Cluster 9, containing 381 genes. **(j)** Plot of Cluster 10, containing 259 genes.

We next apply the proposed HDP clustering algorithm on the yeast cell *Saccharomyces cerevisiae* cycle dataset [2,40]. The data are obtained by synchronizing and collecting the mRNAs from cells at 10-min intervals over the course of two cell cycles. It has been used widely for testing the performances of clustering algorithm [2,14,41]. The expression data have been taken logarithmic transform and lie in the interval [−2,2]. We pre-processed the data to remove those which did not change significantly over time. We also removed those data whose means are below a small threshold. After the pre-processing, there are 1,515 genes left. We then apply the HDP algorithm and obtain 10 clusters in total. The plots of the clusters are shown in Figure 5.

We resort to the MIPS database [42] to determine the functional categories for each cluster. The inferred functional category of a cluster is the category shared by the majority of the member elements. After applying the cell-cycle selection criterion in [2], we find that there are 126 genes identified by proposed HDP algorithm but not discovered in [2]. We list in Table 5 the numbers of newly discovered genes in various functional categories. We also observe that parts of the newly discovered unclassified genes belong to clusters with classified categories. Given the hierarchical characteristic of the HDP algorithm, it may suggest multiple descriptions of those genes that might have been overlooked before.

**Table 5:** Numbers of newly discovered genes in various functional categories by the proposed HDP clustering algorithm

**Function categories**	**Number of newly discovered genes**
Cell cycle and DNA processing	20
Protein synthesis	25
Protein fate	4
Cell fate	12
Transcription	8
Unclassified protein	57

Note that in [14] a Bayesian model with infinite number of clusters is proposed based on the Dirichlet process. The model in [14] is a special case of the HDP model proposed in this article when there is only one hierarchy. In terms of discovering new gene functionalities, we find that the performances of the two algorithms are similar, as the method in [14] discovered 106 new genes compared to the result in [2]. However, by taking the hierarchical structure into account, the total number of clusters found by the HDP algorithm is significantly smaller than that given in [14] which is 43 clusters. The SI score for BIMC and HDP are 0.321 and 0.392, respectively. The HDP clustering consolidates many fragmental clusters, which may provide an easier way to interpret the clustering results.

In Table 6, we list the new genes discovered by the HDP algorithm which are not found in [2].

**Table 6:** List of newly discovered genes in various functional categories

**Function categories**	**Genes**
	YBL051c YBR136w YBL016w YDR200c YBR274w
Cell cycle and DNA	YDR217c YLR314c YJL074c YJL095w YDR052c
processing	YDL126c YCL016c YDL188c YAL040c YEL019c
	YER122c YLR035c YLR055c YML032c YMR078c
Protein synthesis	YDR091c YGL103w YBR118w YBL057c YBR101c
	YBR181c YDL083c YDL184c YDR012w YDR172w
	YGL105w YGL129c YJL041w YJL125c YJR113c
	YLR185w YPL037c YPL048w YLR009w YHL001w
	YHL015w YHR011w YHR088w YDR450w YEL034w
Protein fate	YAL016w YBL009w YBR044c YDL040c
Cell fate	YAL040c YDL006w YDL134c YIL007c YJL187c
	YDL029w YDL035c YCR002c YBL105c YCR089w
	YER114c YEL023c
Transcription	YAL021c YBL022c YCL051w YDR146c YIL084c
	YJL127c YJL164c YJL006c

## 5 Conclusions

In this article, we have proposed a new clustering approach based on the HDP. The HDP clustering explicitly models the hierarchical structure in the data that is prevalent in biological data such as gene expressions. We have developed a statistical inference algorithm for the proposed HDP model based on the Chinese restaurant metaphor and the Gibbs sampler. We have applied the proposed HDP clustering algorithm to both regulatory network segmentation and gene expression clustering. The HDP algorithm is shown to reveal more structural information of the data compared to popular algorithms such as SVM and MCLUST, by incorporating the hierarchical knowledge into the model.

## Appendix

### Derivation of formula (19) and (21)

(32) $$\begin{array}{lll} & P\left(\phi_{\text{ji}}=a|\phi_{\text{ji}}^{c},\lambda ,\theta ,\alpha_{1},\alpha_{0},\mu_{1},g\right)\; & \;\\ & \;=\frac{P\left(g_{\text{ji}},\phi_{\text{ji}}=a|\phi^{c}(z_{\text{ji}}),\lambda ,\theta ,\alpha_{1},\alpha_{0},\mu_{1},g_{\text{ji}}^{c}\right)}{P\left(g_{\text{ji}}|\phi_{\text{ji}}^{c},\lambda ,\theta ,\alpha_{1},\alpha_{0},\mu_{1},g_{\text{ji}}^{c}\right)}\; \end{array}$$

(33) $$\begin{array}{ll} & \;\propto P\left(g_{\text{ji}},\phi_{\text{ji}}=a|\phi_{\text{ji}}^{c},\lambda ,\theta ,\alpha_{1},\alpha_{0},\mu_{1},g_{\text{ji}}^{c}\right)\; \end{array}$$

(34) $$\begin{array}{lll} & \;\propto P\left(g_{\text{ji}}|\phi ,\lambda ,\theta ,\alpha_{1},\alpha_{0},\mu_{1},g_{\text{ji}}^{c}\right)\; & \;\\ & \;\;\;P\left(\phi_{\text{ji}}=a|\phi_{\text{ji}}^{c},\lambda ,\theta ,\alpha_{1},\alpha_{0},\mu_{1},g_{\text{ji}}^{c}\right)\; \end{array}$$

By (16), if *a* has appeared before, we have

(35) $$P\left(\phi_{\text{ji}}=a|\phi_{\text{ji}}^{c},\lambda ,\theta ,\alpha_{1},\alpha_{0},\mu_{1},g_{\text{ji}}^{c}\right)\propto c_{\text{ja}}.$$

Otherwise we have

(36) $$P\left(\phi_{\text{ji}}=a|\phi_{\text{ji}}^{c},\lambda ,\theta ,\alpha_{1},\alpha_{0},\mu_{1},g_{\text{ji}}^{c}\right)\propto \alpha_{0}.$$

If *a* has appeared before, by the assumption the data are conditionally independent, we also have

(37) $$P\left(g_{\text{ji}}|\phi ,\lambda ,\theta ,\alpha_{1},\alpha_{0},\mu_{1},g_{\text{ji}}^{c}\right)=f_{\lambda_{\text{ja}}}\left(g_{\text{ji}}|g_{\text{ji}}^{c}\right),$$

where $f_{\lambda_{\text{ja}}}(g_{\text{ji}}|g_{\text{ji}}^{c})$ can be calculated by the Bayes’ formula:

(38) $$f_{\lambda_{\text{ja}}}\left(g_{\text{ji}}|g_{\text{ji}}^{c}\right)=\frac{\int \underset{\lambda_{j^{\prime }\phi_{j^{\prime }i^{\prime }}}=\lambda_{\text{ja}}}{\prod }F(g_{j^{\prime }i^{\prime }}|\theta )\mu_{1}(\theta )\mathrm{d\theta}}{\int \underset{(j^{\prime },i^{\prime })\neq (j,i),\lambda_{j^{\prime }\phi_{j^{\prime }i^{\prime }}}=\lambda_{\text{ja}}}{\prod }F(g_{j^{\prime }i^{\prime }}|\theta )\mu_{1}(\theta )\mathrm{d\theta}}.$$

Combining (35) and (37), we have (19).

If *a* has not appeared before, by (17), we have

(39) $$\;\begin{array}{l} P\left(g_{\text{ji}}|\phi ,\lambda ,\theta ,\alpha_{1},\alpha_{0},\mu_{1},g_{\text{ji}}^{c}\right)\\ =\;\overset{K_{\text{ja}}}{\underset{k=1}{\sum }}\frac{\sum_{j}d_{\text{jk}}}{\sum_{\text{jk}}d_{\text{jk}}\;+\;\alpha_{1}}f_{k}\left(g_{\text{ji}}|g_{\text{ji}}^{c}\right)\;+\;\frac{\alpha_{1}}{\sum_{\text{jk}}d_{\text{jk}}\;+\;\alpha_{1}}\;\;\int \;\;F(g_{\text{ji}}|\theta )\mu_{1}(\theta )\mathrm{d\theta}, \end{array}$$

Combining (36) and (39), we have (21).

### Derivation of (22) nd (23)

(40) $$\begin{array}{lll} & P\left(\lambda_{j\phi_{\text{ji}}}=a|\phi ,\lambda_{j\phi_{\text{ji}}}^{c},\theta ,\alpha_{1},\alpha_{0},\mu_{1},g\right)\; & \;\\ & \;=\frac{P\left(g_{\text{ji}},\lambda_{j\phi_{\text{ji}}}=a|\phi ,\lambda_{j\phi_{\text{ji}}}^{c},\theta ,\alpha_{1},\alpha_{0},\mu_{1},g_{\text{ji}}^{c}\right)}{P\left(g_{\text{ji}}|\phi ,\lambda ,\theta ,\alpha_{1},\alpha_{0},\mu_{1},g_{\text{ji}}^{c}\right)}\; \end{array}$$

(41) $$\begin{array}{ll} & \;\propto P\left(g_{\text{ji}},\lambda_{j\phi_{\text{ji}}}=a|\phi ,\lambda_{j\phi_{\text{ji}}}^{c},\theta ,\alpha_{1},\alpha_{0},\mu_{1},g_{\text{ji}}^{c}\right)\; \end{array}$$

(42) $$\begin{array}{lll} & \;\propto P\left(g_{\text{ji}}|\phi ,\lambda ,\theta ,\alpha_{1},\alpha_{0},\mu_{1},g_{\text{ji}}^{c}\right)\; & \;\\ & \;\;P\left(\lambda_{j\phi_{\text{ji}}}=a|\phi ,\lambda_{j\phi_{\text{ji}}}^{c},\theta ,\alpha_{1},\alpha_{0},\mu_{1},g_{\text{ji}}^{c}\right)\; \end{array}$$

By (17), if *a* has appeared before, we have

(43) $$P\left(\lambda_{j\phi_{\text{ji}}}=a|\phi ,\lambda_{j\phi_{\text{ji}}}^{c},\theta ,\alpha_{1},\alpha_{0},\mu_{1},g_{\text{ji}}^{c}\right)\propto \sum_{j}d_{\text{ja}}.$$

Otherwise we have

(44) $$P\left(\lambda_{j\phi_{\text{ji}}}=a|\phi ,\lambda_{j\phi_{\text{ji}}}^{c},\theta ,\alpha_{1},\alpha_{0},\mu_{1},g_{\text{ji}}^{c}\right)\propto \alpha_{1}.$$

If *a* is used before, we have

(45) $$P\left(g_{\text{ji}}|\phi ,\lambda ,\theta ,\alpha_{1},\alpha_{0},\mu_{1},g_{\text{ji}}^{c}\right)=f_{a}\left(g_{\text{ji}}|g_{\text{ji}}^{c}\right).$$

Otherwise, the customer chooses a new table. The data are generated from *F* based on a sample from *μ*_1_. We have

(46) $$P\left(g_{\text{ji}}|\phi ,\lambda ,\theta ,\alpha_{1},\alpha_{0},\mu_{1},g_{\text{ji}}^{c}\right)=\int F(g_{\text{ji}}|\theta )\mu_{1}(\theta )\mathrm{d\theta .}$$

Combining (43), (44), (45), and (46), we have (22) and (23).

## Competing interests

The authors declare that they have no competing interests.
